# Metabolomics reveals the effects of hydroxysafflor yellow A on neurogenesis and axon regeneration after experimental traumatic brain injury

**DOI:** 10.1080/13880209.2023.2229379

**Published:** 2023-07-07

**Authors:** En Hu, Teng Li, Zhilin Li, Hong Su, Qiuju Yan, Lei Wang, Haigang Li, Wei Zhang, Tao Tang, Yang Wang

**Affiliations:** a Department of Integrated Traditional Chinese and Western Medicine, Institute of Integrative Medicine, Xiangya Hospital, Central South University, Changsha, PR China; b National Clinical Research Center for Geriatric Disorders, Xiangya Hospital, Central South University, Changsha, PR China; c Department of Respiratory Diseases, Xiangxiang People’s Hospital, Xiangxiang, PR China; d Hunan Key Laboratory of the Research and Development of Novel Pharmaceutical Preparations, Changsha Medical University, Changsha, PR China; e The College of Integrated Traditional Chinese and Western Medicine, Hunan University of Chinese Medicine, Changsha, PR China

**Keywords:** Network pharmacology, brain-derived neurotrophic factor, signal transducer and activator of transcription 3, growth-associated protein 43, cortex, hippocampus, traditional Chinese medicine

## Abstract

**Context:**

Hydroxysafflor yellow A (HSYA) is the main bioactive ingredient of safflower (*Carthamus tinctorius* L., [Asteraceae]) for traumatic brain injury (TBI) treatment.

**Objective:**

To explore the therapeutic effects and underlying mechanisms of HSYA on post-TBI neurogenesis and axon regeneration.

**Materials and methods:**

Male Sprague-Dawley rats were randomly assigned into Sham, controlled cortex impact (CCI), and HSYA groups. Firstly, the modified Neurologic Severity Score (mNSS), foot fault test, hematoxylin-eosin staining, Nissl’s staining, and immunofluorescence of Tau1 and doublecortin (DCX) were used to evaluate the effects of HSYA on TBI at the 14th day. Next, the effectors of HSYA on post-TBI neurogenesis and axon regeneration were screened out by pathology-specialized network pharmacology and untargeted metabolomics. Then, the core effectors were validated by immunofluorescence.

**Results:**

HSYA alleviated mNSS, foot fault rate, inflammatory cell infiltration, and Nissl’s body loss. Moreover, HSYA increased not only hippocampal DCX but also cortical Tau1 and DCX following TBI. Metabolomics demonstrated that HSYA significantly regulated hippocampal and cortical metabolites enriched in ‘arginine metabolism’ and ‘phenylalanine, tyrosine and tryptophan metabolism’ including l-phenylalanine, ornithine, l-(+)-citrulline and argininosuccinic acid. Network pharmacology suggested that neurotrophic factor (BDNF) and signal transducer and activator of transcription 3 (STAT3) were the core nodes in the HSYA-TBI-neurogenesis and axon regeneration network. In addition, BDNF and growth-associated protein 43 (GAP43) were significantly elevated following HSYA treatment in the cortex and hippocampus.

**Discussion and conclusions:**

HSYA may promote TBI recovery by facilitating neurogenesis and axon regeneration through regulating cortical and hippocampal metabolism, BDNF and STAT3/GAP43 axis.

## Introduction

Traumatic brain injury (TBI) is a major cause of global death (Rubin 2020). In addition, most survivors experience prolonged sequelae (Mccrea et al. 2021). The functional sequelae primarily result from neuronal loss and dysfunction. Currently, two strategies are mainly adopted for TBI treatment: neuroprotection in the acute stage and neural restoration in the subacute stage (Xiong et al. 2013; Meyfroidt et al. 2022). However, no effective neuroprotective agents are available in TBI treatment due to the limited therapeutic window in the acute stage (Xiong et al. 2013; Jarrahi et al. 2020). Thus, it is pivotal to facilitate neural restoration for the long-term recovery of TBI (Xiong et al. 2013; Jarrahi et al. 2020; Redell et al. 2020; Meyfroidt et al. 2022).

Neurogenesis and axon regeneration are the main processes in neural restoration (Chen et al. 2014; Zhang, Bogdanova, et al. 2014; Zhang, Yu, et al. 2014). Neurogenesis supplements the lost neurons (Chen et al. 2014). Axon regeneration can re-establish the communicating network in the injured brain (Zhang, Bogdanova, et al. 2014; Zhang, Yu, et al. 2014). They aid functional reconstruction following TBI (He et al. 2020; Redell et al. 2020). However, the intrinsic extents of neurogenesis and axon regeneration need to be improved for overall functional improvement after TBI (Jarrahi et al. 2020). In the adult mammal brain, the mature neuron cannot proliferate, and neurogenesis is restricted to the subventricular zone and the subgranular zone of the hippocampus (Redell et al. 2020). Axon regeneration in the adult central nervous system (CNS) is also limited, even following the injury stimuli (Ribas et al. 2021). Thus, it is valuable to strengthen neurogenesis and axon regeneration for TBI recovery.

As the main active ingredient and quality marker of safflower (*Carthamus tinctorius* L., [Asteraceae]), hydroxysafflor yellow A (HSYA) is a widely prescribed traditional Chinese medical component for treating ‘blood stagnation syndrome’ (Bai et al. 2020; Chinese Pharmacopoeia Commission 2020). Earlier studies have reported the antioxidative, free radical-scavenging and anti-inflammatory activities of HSYA in cardiovascular diseases, diabetes and neurological diseases (Pei et al. 2017; Bai et al. 2020; Lee et al. 2020). In experimental TBI, HSYA is primarily administered in the hyperacute and acute stages to protect brains from inflammation and oxidative stress-induced secondary brain injury (Bie et al. 2010; Wang et al. 2016; Li et al. 2021; Xu et al. 2021). However, several lines of evidence have indicated that HSYA may also assist in TBI restoration. First, HSYA can increase brain-derived neurotrophic factor (BDNF) in rat brains. The latter is one of the critical regulators of CNS neurogenesis and axon regeneration (Xing et al. 2016). Second, this component inhibits excessive fibre deposition (Liu et al. 2014; Pan et al. 2017), which forms a scar to hamper axon growth after CNS trauma (Huebner and Strittmatter 2009; Ayazi et al. 2022). Additionally, in the theory of traditional Chinese medicine, ‘blood stagnation syndrome’ targeted by HSYA exists throughout all stages of TBI (Shu et al. 2008). Therefore, we speculate that HSYA may promote neurogenesis and axon regeneration after TBI besides neuroprotection.

Metabolomics is a high-throughput technology to detect small molecule metabolites in the biological system (Kaddurah-Daouk et al. 2008). It is widely applied to reflect disease-associated and drug-responsive metabolic alterations (Li et al. 2020, 2021). As functional molecules, the altered metabolites tend to explain the therapeutic effects of drugs. Besides, metabolomics is often integrated with network pharmacology to uncover the underlying mechanisms (Li et al. 2021). Previously, a metabolomics and network pharmacology-based study explored the neuroprotective mechanisms of HSYA on TBI in the acute stage. This study unveiled that HSYA alleviated post-TBI brain injury through its anti-inflammatory and antioxidative activities through normalizing hypoxanthine-xanthine balance and down-regulating arachidonic acid (Li et al. 2021). However, this published study just focused on the acute stage. In this stage, neurogenesis and axon regeneration are not apparent (Redell et al. 2020). Thus, the corresponding metabolic changes may be masked. Besides, in the network pharmacology scheme, the authors intersected the potential HSYA targets with all TBI targets and highlighted the hub genes. Therefore, this strategy may miss many less attended but equally important effectors.

Therefore, in this study, we evaluated the effects of HSYA on neurogenesis and axon regeneration following TBI at the subacute stage. To find the potential targets of HSYA in promoting post-TBI neurogenesis and axon regeneration, we screened the HSYA-responsive metabolites in the hippocampus and cortex at the subacute stage by untargeted metabolomics. Moreover, a pathology-specialized network pharmacology strategy was integrated to extract the effectors of HSYA on post-TBI neurogenesis and axon regeneration. In addition, the core effectors were further validated. Based on the specialized strategy, we discovered novel effectors of HSYA, including growth-associated protein 43 (GAP43), which is closely related to axon outgrowth. The results will extend our understanding of the potential effective windows of HSYA on TBI and uncover the mechanism of HSYA on TBI recovery.

## Materials and methods

### Animals

Male Sprague-Dawley rats (8 weeks, 220–250 g) were obtained from the Hunan Slake Jingda Laboratory Animal Co., Ltd. (Changsha, China). They were housed according to the Guide for the Care and Use of Laboratory Animals of the National Institutes of Health (NIH Publication No. 85-23, revised 1996). In addition, all experimental protocols were approved by the Medical Ethics Committee of Central South University (NO. 201803421).

### Experimental design and drug administration

According to the purpose and sample processing, the animal study was divided into two sequential parts:

Part 1. To evaluate the effects of HSYA in post-TBI neurogenesis and axon regeneration, 15 rats were randomly assigned into Sham, controlled cortex impact (CCI), and HSYA groups (*n* = 5 in each group). The TBI model was induced by CCI. The Sham rats underwent the same surgery as CCI but without impact. The HSYA (13.88 mg/kg, purity 98%, cat: B20968, lot: R19O11F127953, YuanYe Biotechnology Co., Ltd. Shanghai, China) was intra-gastrically administered daily for 14 d after CCI (Chinese Pharmacopoeia Commission 2020; Li et al. 2021). An equal volume of distilled water was given to the Sham and the CCI rats similarly. The modified Neurologic Severity Score (mNSS), foot fault rate and weight changes were recorded. On the 14th day after surgery, rats were sacrificed and the brains were fixed in 4% paraformaldehyde after normal saline perfusion. The samples were used for histological examination and the latter core-effectors validation.
Part 2. To detect the HSYA-induced metabolic responses in the subacute stage of TBI, 30 rats were grouped and treated as Part 1 did (*n* = 10 in each group). Again, the mNSS and body weight were assessed. After 14 d, rats were perfused with normal saline. The peri-injured cortex and hippocampus were harvested and snap-frozen in liquid nitrogen for metabolomic detection.

A total of 48 rats were used. In Part 1, all rats survived until being sacrificed. In Part 2, one rat died due to an unanticipated anaesthesia event, and two deaths occurred immediately after TBI induction but before HSYA (or solvent) administration. They were later replaced with other rats. All survival animals were included for further analyses.

### TBI induction

As previously described, TBI was established by the CCI method (Li et al. 2021). In brief, rats were anesthetized with 3% pentobarbital sodium (60 mg/kg, i.p.). The skin and skull (5.0 mm diameter) above the right parietal cortex (the centre coordinates were 3.0 mm posterior, 3.0 mm lateral to bregma) were removed in sequence. CCI impact parameters were as follows: impact velocity (6.0 m/s), impact depth (5.0 mm) and contact time (50 ms). The skin was then closed. The rats’ body temperature was monitored and maintained at 37.0 ± 0.5 °C.

### Neurobehavior test

The mNSS test, foot fault test and weight changes were evaluated on days 0, 1, 3, 7 and 14 after CCI. The assessment of mNSS includes the motor, sensory, reflex and balance abilities. The grades ranged from 0 to 18. Higher scores represent worse performance. The foot fault test recorded the number of forepaws falling when rats walked on the grid within 1 min. Foot fault rates were calculated as (contralateral foot faults-ipsilateral foot faults)/total steps of forepaws 100%. The neurobehaviour parameters were judged by two researchers who were blind to the experimental groups independently.

### Histopathology and immunohistochemistry examination

Brains were fixed in 4% paraformaldehyde for 24 h and cut into 3.0 μm coronal paraffin sections. For hematoxylin and eosin (H&E) staining, the rehydrated sections were immersed in hematoxylin (G1004, Servicebio, Wuhan, China) and eosin Y (G1001, Servicebio) for 5 min and 20 s, respectively. For Nissl’s staining, the slices were incubated in the toluidine blue solution (G1036, Servicebio) for 8 min. For immunofluorescent, 3% bovine serum albumin in 0.02% Triton-X100 was used for blocking. Then, primary antibodies were incubated at 4 °C for 14 h, including rabbit anti-BDNF (ab108319, 1:1000, Abcam, Cambridge, MA), rabbit anti-Tau1 (MAB3420, 1:400, Millipore, Billerica, MA), rabbit anti-GAP43 (8945S, 1:800, Cell Signaling Technology, Danvers, MA), rabbit anti-doublecortin (DCX, 4604S, 1:800, Cell Signaling Technology), mouse anti-proliferating cell nuclear antigen (PCNA, cell proliferation marker, SC-56, 1:1000, Santa Cruz Biotechnology, Dallas, TX). Cy3 or Alex Fluor 488-conjugated secondary antibodies were utilized for visualization. Negative controls were performed by omission of primary antibodies. For each measurement, three slices were stained at 0.5 mm intervals in each animal. The first slice was cut at about −3.0 mm level (relative to bregma), where the structure of the hippocampus and the edge of the wound were typical according to the standard rat stereotaxic atlas (Paxinos and Watson 2007). The experimental groups were blinded to statistic researchers.

### LC-MS analysis

A high-performance liquid chromatography (HPLC, 1260 infinity, Agilent, Santa Clara, CA) coupling mass spectra (MS)/MS (Q-Exactive, Thermofisher, Waltham, MA) was applied for metabolite detection. An amide column was employed for chromatographic separations. The parameters were identical to our previous study (Li et al. 2021).

### Metabolic profiling and pathway analysis

The raw data were entered into Compound Discover 2.1 (Thermofisher). Intensities were rectified by fitting the loess regression model to the quality control (QC) samples. The *α* parameter was set to 2 to prevent overfitting. Then, the features with >80% missing values and relative standard deviations (RSDs) >30% (in QCs) were ruled out. Features were identified by performing retention time (RT) alignment (maximum time shift: 2 min), unknown compound detection (mass tolerance: 10.0 ppm), compound grouping (time tolerance: 0.5 min), and compound annotation (mass tolerance: 5.0 ppm). Features that fully matched the MS2 spectrum in the mzCloud database underwent further analyses. The pretreated data were transformed with log and scaled with Pareto, then analysed using partial least squares discrimination analysis (PLS-DA) in MetaboAnalyst version 5.0 (https://www.metaboanalyst.ca/). The cross-validation and 100-times permutation tests were used to confirm the accuracy. Differential metabolites were screened by variable importance in projection (VIP) >1 and *p* < 0.05 (Student’s *t*-test). Heatmaps and metabolic pathway analysis were processed by the MetaboAnalyst website.

### Metabolite-gene-HSYA network analysis

The metabolite-associated proteins were obtained from STITCH (http://stitch.embl.de/) and PubChem (https://pubchem.ncbi.nlm.nih.gov/). Genes related to neurogenesis and axon regeneration were downloaded from GeneCards (https://www.genecards.org/). The potential targets of HSYA were gathered from SEA (https://sea.bkslab.org/), Swiss target prediction (http://www.swisstargetprediction.ch/), TCMSP (https://tcmsp-e.com/tcmsp.php), Pharmmapper (http://www.lilab-ecust.cn/pharmmapper/), STITCH and PubChem databases. Then, all the gene names were converted into rat homologs by R package ‘Homologene’. Finally, the networks were constructed by Cytoscape version 3.7.2 (Cytoscape Consortium, San Diego, CA).

### Statistical analyses

Statistical analyses were performed by Student’s *t*-test (two-group comparison) or one-way ANOVA followed by Dunnett’s *t*-test (more than two groups, the CCI group as control) using SPSS version 26 (IBM Corp., Armonk, NY). A *p* value of < 0.05 was regarded as statistically significant. Data were expressed as mean ± standard deviation.

## Results

### The effects of HSYA on neurological deficits and brain histopathology after TBI in the subacute stage

**Figure 1.**
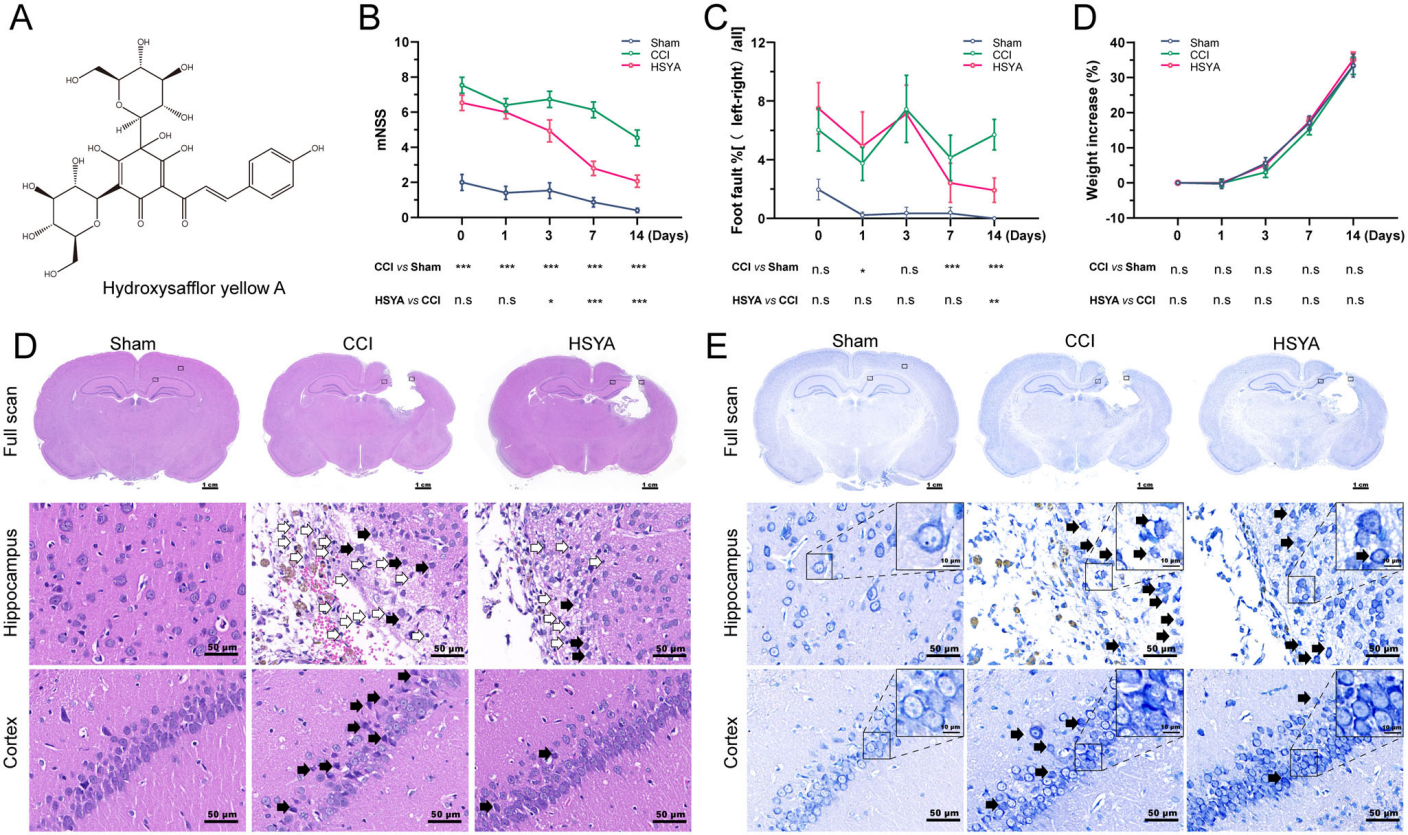
HSYA promotes TBI recovery. (A) The chemical structure of HSYA. (B) HSYA reduces mNSS after TBI. (C) HSYA decreases foot fault rate after TBI in the subacute stage. (D) HSYA tends to improve weight loss after TBI. (E) Representative images of H&E indicate that HSYA normalizes neuron damage and disorganization (black arrow) and decreases inflammatory cells (white arrows) in the cortex and hippocampus. (F) Representative images of Nissl’s staining indicate that HSYA reduces the number of injured neurons in the cortex and the hippocampus. CCI: controlled cortical impact; CTX: cortex; HP: hippocampus; data are expressed as mean ± standard deviation; *n* = 15 (A and C), *n* = 5 (B, D and E); *P* is calculated by one-way ANOVA followed by Dunnett’s t-test; *p < 0.05; **p < 0.01; ***p < 0.001.

The chemical structure of HSYA is shown in Figure 1(A). On the day after surgery and before drug administration (day 0), the mNSS (Figure 1(B)) and foot fault rate (Figure 1(C)) in the CCI group were significantly higher than that of the Sham group. The significance existed until the 14th day. Compared with the CCI group, HSYA markedly reduced the mNSS (Figure 1(B)) and foot fault rate (Figure 1(C)) since day three after TBI. The changes in body weight were not evident among the three groups in the experimental time points (Figure 1(D)).

H&E showed that HSYA ameliorated post-TBI neuron damage (black arrows), reversed tissue and cell disorganization and decreased inflammatory cell infiltration (white arrows) in the cortex and hippocampus (Figure 1(E)). In addition, Nissl’s staining suggested that HSYA improved neuron injury and disarrangement (Figure 1(F), black arrows).

### The effects of HSYA on neurogenesis and axon regeneration in the subacute stage of TBI

**Figure 2.**
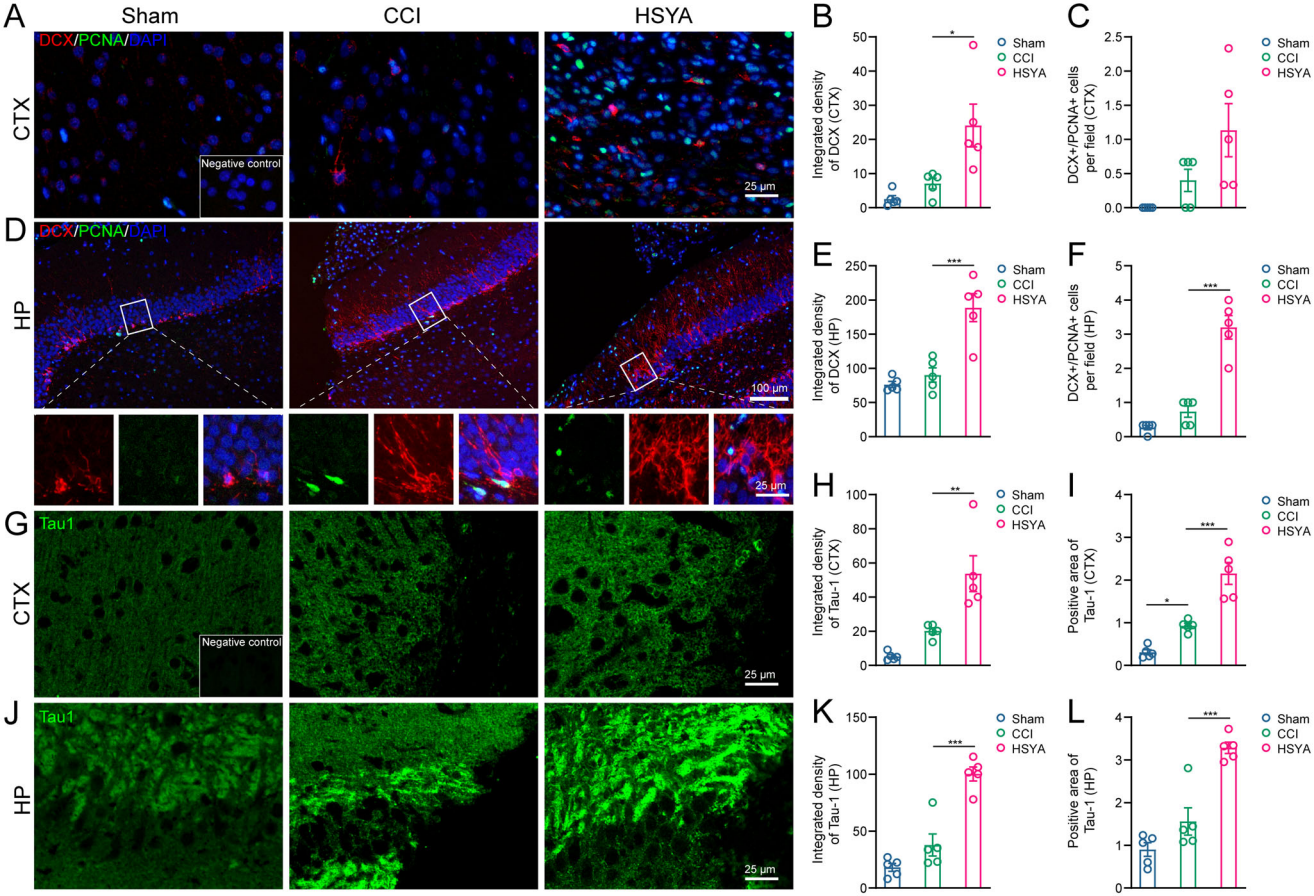
HSYA facilitates neurogenesis and axon regeneration. (A) Immunofluorescent staining of DCX (red) and PCNA (green) indicates more DCX^+^ immature neurons but not DCX^+^/PCNA^+^ proliferating neurons in the HSYA group in the cortex. (B) The statistical graph shows an increased density of DCX in the cortex after HSYA treatment. (C) The statistical graph displays no significance between CCI and HSYA groups on the number of DCX^+^/PCNA^+^ proliferating neurons. (D) Immunofluorescent indicates more immature neurons proliferating neurons in the HSYA group in the hippocampus. (E) HSYA increases the density of DCX in the hippocampus. (F) HSYA increases the number of proliferating neurons in the hippocampus. (G) Immunofluorescent staining of Tau1 (green) indicates more axons in the HSYA group in the cortex around the wound. (H) The statistical graph shows the increased integrative density of Tau1 in the cortex after HSYA treatment. (I) The statistical graph displays an elevated positive area of Tau1 in the HSYA group than in the CCI one. (J) Immunofluorescence shows more axons in the HSYA group in the hippocampus after TBI. (K) HSYA increases the integrative density of Tau1 in the hippocampus. (L) HSYA elevates the positive area of Tau1 in the hippocampus. Data are expressed as mean ± standard deviation; *n* = 5 (A–I); *P* is calculated by one-way ANOVA followed by Dunnett’s *t*-test; *p < 0.05; **p < 0.01; ***p < 0.001.

Neurogenesis was accessed by double staining of DCX and PCNA. In the cortex, the DCX-positive immature neurons seldom appeared in the Sham and CCI groups, but the density of DCX signals was significantly enhanced after HSYA treatment (Figure 2(A,B)). However, the number of newborn neurons (DCX^+^ and PCNA^+^ cells) in the HSYA group was not significantly changed compared with CCI rats (Figure 2(C)). In the hippocampus, both immature and newborn neurons were augmented considerably by HSYA (Figure 2(D–F)).

A fluorescent signal of Tau1 protein was adopted to reflect the levels of axon regeneration. While the core of the injury was negatively stained, the peri-injured area of the cortex and the hippocampus showed intensified Tau1 expression after TBI. Furthermore, after HSYA treatment, the integrated density and positive area of Tau1 were significantly increased (Figure 2(G–L)).

### The effects of HSYA on cortical and hippocampal metabolic profiles in the subacute stage of TBI

**Figure 3.**
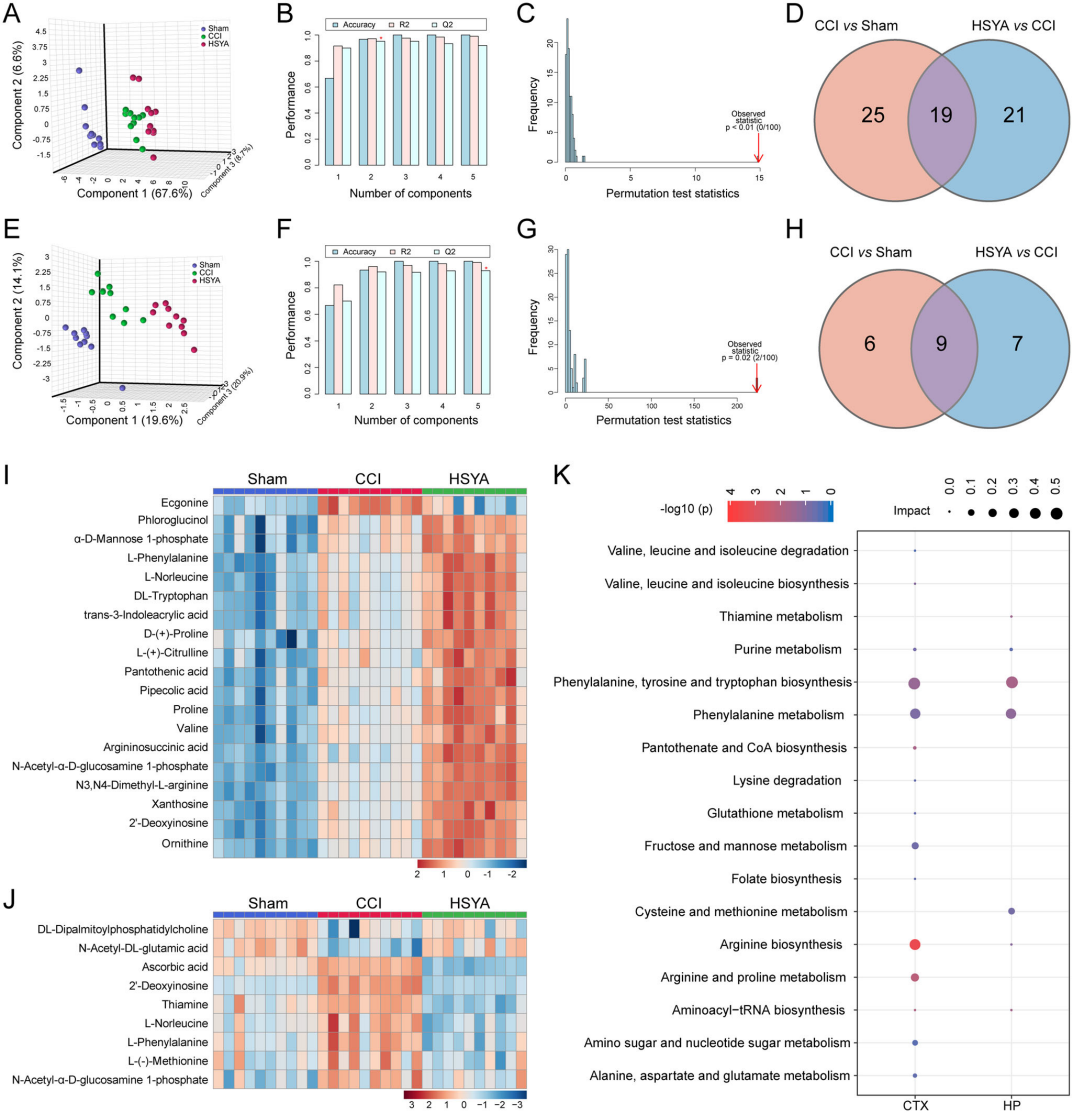
HSYA regulates the metabolic pattern of the post-TBI cortex and hippocampus. (A) PLS-DA plot shows distinct metabolic differences among Sham, CCI and HSYA groups in the cortex. (B) The cross-validation graph shows favourable reliability and predictability with high accuracy (1), R2 (0.9898) and Q2 (0.91921) in the cortex. (C) The 100-times permutation test indicates appreciable discriminability and adaptability of the PLS-DA model in the cortex with *p* < 0.05. (E) PLS-DA plot shows distinct metabolic differences among Sham, CCI and HSYA groups in the hippocampus. (F) The cross-validation graph shows favourable reliability and predictability with high accuracy (1), R2 (0 0.99135) and Q2 (0.9292) in the hippocampus. (G) The 100-times permutation test indicates appreciable discriminability and adaptability of the PLS-DA model in the hippocampus with *p* < 0.05. (D) Venn graph shows HSYA regulates 19 metabolites in the post-TBI cortex. (H) Venn graph suggests HSYA regulates nine metabolites in the post-TBI hippocampus. (I) Heatmap displays the expression levels of significantly changed metabolites in the cortex. (J) The expression levels HSYA responsive metabolites in the hippocampus. (K) The related metabolic pathway in the cortex and the hippocampus. *N* = 10 (A–K).

The 3D PLS-DA suggested that samples were clustered closely within the same group but separated among groups in the cortex (Figure 3(A)) and hippocampus (Figure 3(E)). The cross-validation showed favourable reliability and predictability with high accuracy (1, 1), *R*2 (0.9898, 0.99135) and *Q*2 (0.91921, 0.9292) in the cortex (Figure 3(B)) and hippocampus (Figure 3(F)), respectively. Furthermore, the *p*-value of 100-times permutation tests was less than 0.05, which indicated the appreciable discriminability and adaptability of the PLS-DA model (Figures 3(C,G)).

By integrating VIP >1 and *p* < 0.05, we identified 19 and 9 metabolites as HSYA-responsive metabolites in the subacute stage of TBI in the cortex (Figure 3(D) and hippocampus (Figure 3(H)), respectively (Table 1). Most significantly changed metabolites were upregulated in the cortex after TBI and further enhanced after HSYA treatment (Figure 3(I)). In contrast, in the hippocampus, most metabolites were increased due to TBI but decreased by HSYA (Figure 3(J)).

**Table 1.** Significantly changed metabolites in after HSYA treatment in TBI brains.

Regions	Metabolites	MW	*m/z*	RT (min)	Adducts	HMDB ID	TBI *vs.* Sham			HYSA *vs.* TBI		
							*P* ^a^	VIP^b^	FC^c^	*P*	VIP	FC
CTX	2′-Deoxyinosine	252.08591	251.07885	2.544	[M − H]	HMDB0000071	1.77E-02	1.15	2.29	2.61E-07	1.13	2.43
CTX	Argininosuccinic acid	290.12212	291.12943	20.828	[M + H]	HMDB0000052	1.99E-02	1.04	1.73	2.75E-08	1.26	2.76
CTX	D-(+)-Proline	115.06342	116.07077	21.783	[M + H]	HMDB0003411	2.74E-03	1.03	1.61	2.11E-07	1.17	1.77
CTX	DL-Tryptophan	204.08947	203.08219	6.658	[M − H]	HMDB0013609	1.14E-02	1.04	1.65	1.68E-05	1.03	1.72
CTX	Ecgonine	185.1047	186.1123	2.157	[M − H]	HMDB0006548	3.04E-08	1.34	4.95	6.97E-08	1.19	0.30
CTX	L-(+)-Citrulline	175.09546	176.10274	18.952	[M + H]	HMDB0000904	2.14E-03	1.02	1.57	2.58E-05	1.05	1.53
CTX	L-Norleucine	131.09447	132.10174	6.088	[M + H]	HMDB0001645	3.78E-03	1.08	1.74	3.13E-06	1.08	1.76
CTX	L-Phenylalanine	165.07816	164.07077	5.984	[M − H]	HMDB0000159	6.20E-03	1.09	1.81	2.20E-06	1.10	1.87
CTX	*N*3, *N*4-Dimethyl-L-arginine	202.14271	203.14998	21.479	[M + H]	HMDB0003334	9.17E-05	1.24	2.10	5.16E-08	1.17	1.87
CTX	*N*-Acetyl-α-D-glucosamine 1-phosphate	301.05662	300.04951	20.567	[M-H]	HMDB0001367	2.49E-04	1.26	2.23	2.75E-08	1.28	2.45
CTX	Ornithine	132.08983	133.09711	21.783	[M + H]	HMDB0000214	1.99E-04	1.24	1.54	3.07E-08	1.22	1.69
CTX	Pantothenic acid	219.11045	220.11767	5.003	[M + H]	HMDB0000210	3.16E-03	1.12	1.56	3.29E-05	1.04	1.54
CTX	Phloroglucinol	126.0317	127.039	20.845	[M + H]	HMDB0002215	4.46E-05	1.08	1.83	1.24E-04	1.05	1.42
CTX	Pipecolic acid	129.07884	130.08621	22.086	[M + H]	HMDB0000070	1.44E-03	1.12	1.53	2.18E-07	1.15	1.66
CTX	Proline	115.06347	116.07075	9.077	[M + H]	HMDB0000162	2.95E-03	1.18	1.78	3.47E-07	1.12	1.87
CTX	*trans*-3-Indoleacrylic acid	187.06303	188.07031	6.657	[M + H]	HMDB0000734	1.28E-02	1.01	1.67	1.64E-05	1.02	1.75
CTX	Valine	117.07908	118.08635	8.850	[M + H]	HMDB0000883	3.97E-04	1.17	1.73	1.23E-05	1.07	1.55
CTX	Xanthosine	284.07595	283.06881	3.293	[M − H]	HMDB0000299	8.54E-04	1.28	2.64	2.75E-08	1.31	3.14
CTX	α-D-Mannose 1-phosphate	260.02975	261.03656	20.839	[M − H]	HMDB0006330	9.59E-05	1.10	1.77	2.77E-05	1.07	1.48
HP	2′-Deoxyinosine	252.08591	251.07885	2.544	[M − H]	HMDB0000071	2.78E-08	3.49	10.97	2.77E-08	3.43	0.06
HP	Ascorbic acid	176.03124	175.02396	7.913	[M − H]	HMDB0000044	5.90E-08	2.26	2.87	2.75E-08	3.63	0.05
HP	DL-Dipalmitoylphosphatidylcholine	733.56152	734.56878	1.155	[M + H]	HMDB0245715	1.27E-04	2.22	0.37	3.89E-02	1.34	1.90
HP	L- (-)-Methionine	149.05092	150.0582	7.853	[M + H]	HMDB0000696	1.86E-02	1.04	1.40	5.18E-03	1.07	0.66
HP	L-Norleucine	131.09447	132.10174	6.088	[M + H]	HMDB0001645	3.00E-04	1.18	1.44	4.51E-06	1.38	0.58
HP	L-Phenylalanine	165.07816	164.07077	5.984	[M − H]	HMDB0000159	6.14E-04	1.15	1.43	1.13E-05	1.36	0.59
HP	*N*-Acetyl-DL-glutamic acid	189.06238	188.0551	18.937	[M − H]	HMDB0001138	1.92E-05	1.41	0.64	2.40E-04	1.09	1.47
HP	*N*-Acetyl-α-D-glucosamine 1-phosphate	301.05662	300.04951	20.567	[M − H]	HMDB0001367	2.54E-04	1.52	1.79	4.62E-03	1.06	0.67
HP	Thiamine	264.10414	263.0968	14.517	[M − H]	HMDB0000235	8.24E-05	1.35	1.53	3.13E-08	2.06	0.37

^a^*p* valve from one-way ANOVA; ^b^VIP: Variable importance in the projection from PLS-DA; ^c^FC: fold of change; CTX: cortex; HP: hippocampus.

In the cortex, the HSYA-responsive metabolites were primarily enriched in ‘Arginine biosynthesis,’ ‘Arginine and proline metabolism,’ ‘Phenylalanine, tyrosine and tryptophan biosynthesis’ pathways. The related metabolites were proline, d-proline, l-phenylalanine, ornithine, l-(+)-citrulline and argininosuccinic acid. While in the hippocampus, the differential pathway was the ‘phenylalanine, tyrosine, and tryptophan biosynthesis’ pathway where l-phenylalanine hit (Figure 3(K), Table 2).

**Table 2.** Significantly enriched metabolic pathways after HSYA treatment.

Pathways	Regions	*P*	Impact	Hits
Arginine biosynthesis	Cortex	4.01E-04	0.41	Argininosuccinic acid, Citrulline and Ornithine
Arginine and proline metabolism	Cortex	7.86E-03	0.19	D-Proline, Ornithine and L-Proline
Phenylalanine, tyrosine and tryptophan biosynthesis	Cortex	4.44E-02	0.50	L-Phenylalanine
Phenylalanine, tyrosine and tryptophan biosynthesis	hippocampus	2.11E-02	0.50	L-Phenylalanine

### Integrated analysis of metabolomics with network pharmacology

**Figure 4.**
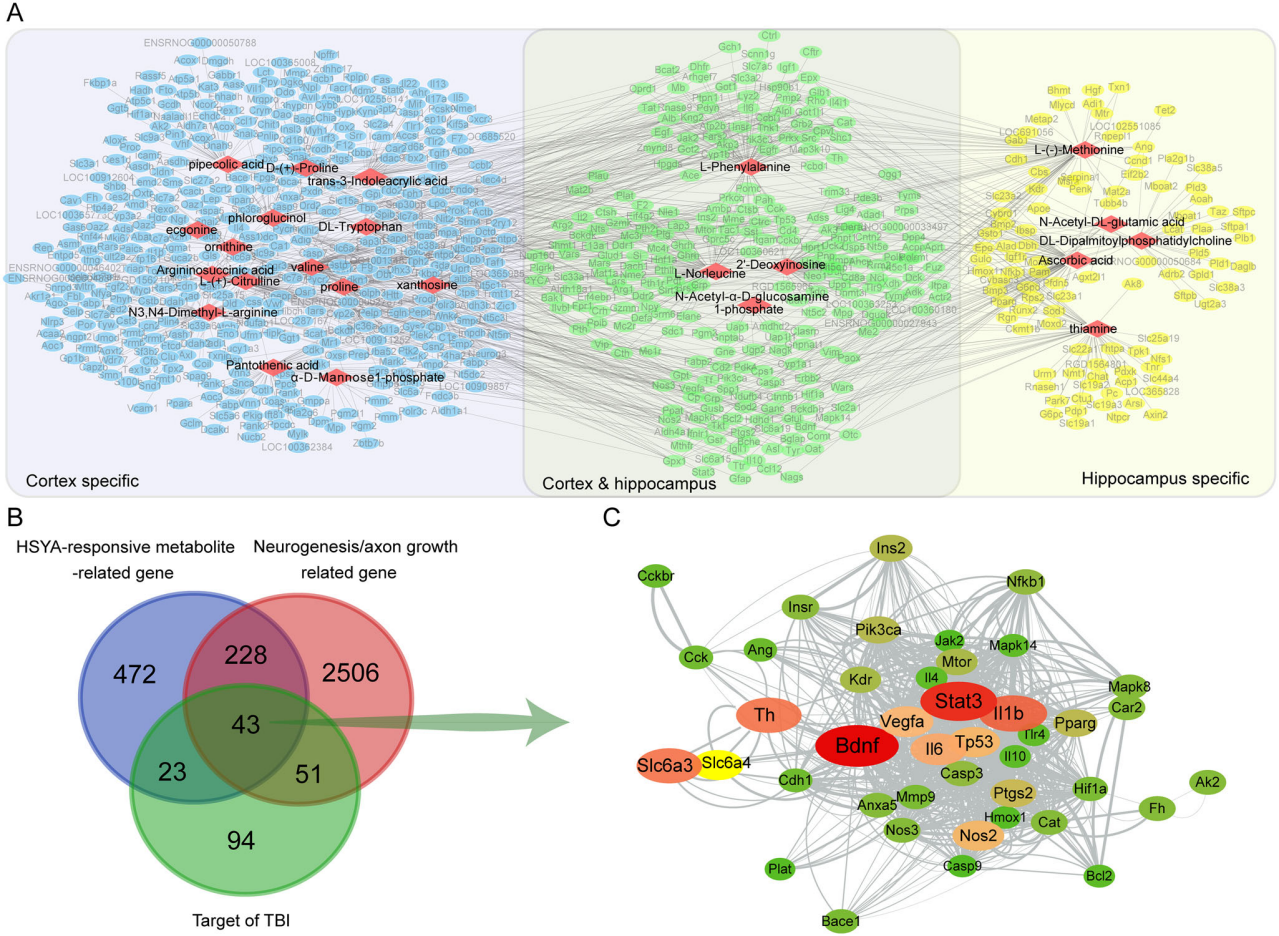
Differential metabolites-directed target exploration of HSYA. (A) Metabolite-gene network shows 24 HSYA-responsive metabolites related to 766 genes; (B) Venn graph indicates 43 genes are shared in the metabolite-related gene, neurogenesis and axon regeneration-related gene, and HSYA-related gene. (C) Protein-protein network indicates a central role of *Bdnf* and *Stat3* in the common genes. The deeper red and larger the ellipse, the closer relevance to neurogenesis and axon regeneration.

Based on the HSYA-responsive metabolites, we further explored the underlying mechanism of HSYA on neurogenesis and axon regeneration. In the metabolite-gene network, 24 metabolites (red diamond) formed 1322 links with 766 genes (ellipse). Among these, 253 genes were related to HSYA-responsive metabolites in the cortex and the hippocampus (green ellipse). At the same time, 412 and 101 genes were cortex (blue ellipse) or hippocampus (yellow ellipse) specific, respectively (Figure 4(A)). Venn diagram displayed that 43 genes were common for the relevant genes of HSYA-responsive metabolites, genes related to neurogenesis and axonal regeneration and targets of TBI. Therefore, they were recognized as the potential effectors of HSYA to promote neural restoration (Figure 4(B)). Furthermore, in the protein-protein network among the shared genes, the genes of BDNF and STAT3 were located near the centre with the highest relevance score to neurogenesis and axon regeneration (Figure 4(C) and Supplementary Table 1).

### The effects of HSYA on BDNF and STAT3/GAP43 axis in the subacute stage of TBI

**Figure 5.**
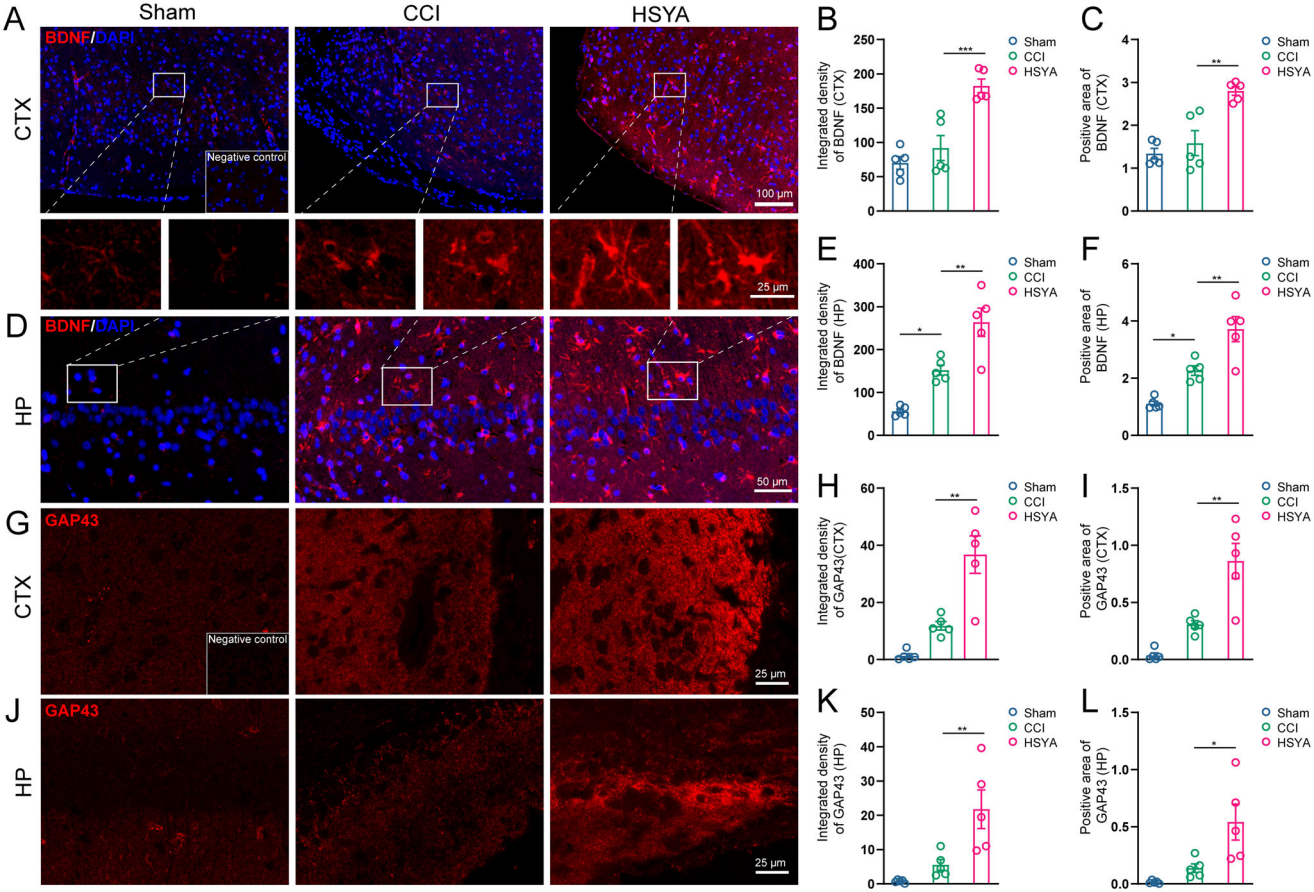
HSYA Increases BDNF and GAP43 expression. (A) Immunofluorescent staining of BDNF (red) indicates more BDNF protein in the HSYA group in the cortex. (B) The statistical graph shows an increased density of BDNF in the cortex after HSYA treatment. (C) The statistical graph shows an increased positive area of BDNF in the cortex after HSYA treatment. (D) Immunofluorescent indicates more BDNF protein in the HSYA group in the hippocampus. (E) HSYA increases the density of BDNF in the hippocampus. (F) HSYA increases the positive area of BDNF in the hippocampus. (G) Immunofluorescent GAP43 (red) staining indicates more GAP43 protein in the HSYA group in the cortex around the wound. (H) The statistical graph shows the increased integrative density of GAP43 in the cortex after HSYA treatment. (I) The statistical graph displays an elevated positive area of GAP43 in the HSYA group than in the CCI one. (J) Immunofluorescence shows more GAP43 in the HSYA group in the hippocampus after TBI. (K) HSYA increases the integrative density of GAP43 in the hippocampus. (L) HSYA elevates the positive area of GAP43 in the hippocampus. Data are expressed as mean ± standard deviation; *n* = 5 (A–I); *P* is calculated by one-way ANOVA followed by Dunnett’s *t*-test; *p < 0.05; **p < 0.01; ***p < 0.001.

Immunofluorescence showed that BDNF was expressed at low levels in the cortex and hippocampus of sham-operated rat brains (Figure 5(A–F)). The level of BDNF tended to increase after TBI in the peri-injured cortex (Figure 5(A–C), *p* > 0.05) and was significantly upregulated in the post-TBI hippocampus (Figure 5(D–F)). HSYA significantly promoted BDNF expression both in the cortex and in the hippocampus. The Sham brains expressed a lower level of GAP43 in the cortex (Figure 5(G–I)) and the hippocampus than that of the CCI group (Figure 5(J–L)). After HSYA treatment, GAP43 expressions tended to increase (*p* > 0.05, Figure 5(G–L)).

## Discussion

TBI is a chronic and lifelong disorder in survivors (Wilson et al. 2017; Meyfroidt et al. 2022). Moreover, the neuropathology of TBI (including axon regeneration and neuron loss) is evolving besides the acute stage, resulting in elevated mortality for many years after TBI (Wilson et al. 2017). Therefore, a neurorepair-targeted therapy is imperative for TBI improvement. This study focused on the subacute stage when the neurorepair is enhanced, but the inflammation and oxidative stress are mitigated (Redell et al. 2020; Fesharaki-Zadeh 2022; Zheng R et al. 2022). We first reported that HSYA enhanced neurogenesis and axon regeneration following TBI. To uncover the potential mechanisms, we integrated metabolomics (cortical and hippocampal) with pathology (neurogenesis and axon regeneration)-specific network pharmacology. Through this approach, we found that HSYA regulated ‘arginine metabolism’, ‘phenylalanine, tyrosine and tryptophan metabolism’ and BDNF expression. These results are consistent with previous studies in acute TBI and vascular dementia (Xing M et al. 2016; Li et al. 2021). Moreover, our study implies a novel mechanism: HSYA may ease axon regeneration by enhancing GAP43 expression. This study suggests that HSYA is a candidate drug for post-TBI neurorepair.

HSYA is derived from safflower, an oral-administered herb (Chinese Pharmacopoeia Commission 2020). A previous study shows that orally-delivered HSYA can be absorbed through the gut and detected in the plasma (Tian et al. 2010). Besides, HSYA can also pass through the injured blood–brain barrier of post-TBI patients (Sheng et al. 2020). In our recent study, oral administration of HSYA effectively improves neurological deficits after TBI (Li et al. 2021). Thus, we treated rats with HSYA orally in this study. According to the Chinese Pharmacopoeia 2020 Edition, the effective dose of safflower is 10 g per day for adults weighing 70 kg, and the required concentration of HSAY in safflower is higher than 1% (Chinese Pharmacopoeia Commission 2020). Therefore, we adopted 13.88 mg/kg in our study. This dose has been reported as effective in TBI treatment (Li et al. 2021). Based on this dose, we found that HSYA alleviated post-TBI neurobehaviour deficits and microstructure abnormalities in the subacute stage. It agrees with the effects of HSYA on acute TBI (Li et al. 2021), vascular dementia (Xing et al. 2016) and ischemic stroke (Yu et al. 2018).

Neurogenesis and axon regeneration contribute to functional recovery after TBI. This study showed insignificant differences of immature neurons and newborn neurons in the hippocampus between the Sham and CCI groups on the 14th day. The results coincided with previous observations indicating impaired neurogenesis until the 14th day after TBI (Rola et al. 2006). However, the density of immature neurons and the number of proliferating immature neurons were significantly increased in the hippocampus after HSYA administration, suggesting enhanced neurogenesis by HSYA. In the mammal brain, the proliferative neural progenitor cells reside only in the subventricular zone and the subgranular zone of the hippocampus. But the immature neurons can migrate to other regions (Redell et al. 2020). This explained our results that the number of immature neurons but not the proliferating neurons was significantly increased in the cortex following HSYA treatment. The CNS axon is hard to regenerate after injury due to intrinsic barriers like astroglia and its extracellular matrix (Burda et al. 2016; Orr and Gensel 2018; Ribas et al. 2021). Thus, the density and positive area of axon marker Tau1 were not enhanced after CCI. HSYA markedly upregulated axon levels around the edge of the wound in the cortex and hippocampus in our study. It suggests that HSYA may be a promising candidate for promoting TBI recovery.

‘Arginine and proline metabolism’ pathway is related to axon regeneration (Zhang et al. 2022). In addition, citrulline and argininosuccinic acid in the ‘arginine biosynthesis’ pathways strongly correlate to neurogenesis in depression (Taniguchi et al. 2021). Mechanically, the enhanced arginine metabolism supplies essential raw materials for axon regeneration and leads to insulin growth factor-1 (IGF-1) upregulation that eases neurogenesis (Taniguchi et al. 2021; Zhang et al. 2022). L-phenylalanine is a critical metabolite in the ‘phenylalanine, tyrosine and tryptophan biosynthesis pathway. In our study, it was markedly increased after TBI in the cortex and the hippocampus. HSYA further elevated l-phenylalanine intensity in the cortex but reduced it in the hippocampus. The discrepancy probably resulted from different morphological and metabolic patterns between the cortex and hippocampus (Hall et al. 2005; Osier et al. 2015; Zheng et al. 2020). Excessive phenylalanine inhibits neurite outgrowth (Hartwig et al. 2006) and induces oxidative stress (Fernandes et al. 2010). Therefore, the overexpressed phenylalanine found in our study may hamper post-TBI neurogenesis and axon regeneration. HSYA normalized the phenylalanine level in the hippocampus, which may create a permissive environment for neural restoration. The presumption is supported by a previous study in ischemic stroke, suggesting that HSYA elicits a neuroprotective effect by downregulating phenylalanine (Chen et al. 2019). However, phenylalanine reduces the viability of astrocytes (Preissler et al. 2016). In the subacute stage of TBI, astrocyte over-proliferates and forms scars around the wound, which obstacles axon elongation and neuron migration (Chen et al. 2019; Michinaga and Koyama Y 2021). Thus, the HSYA-augmented phenylalanine in the cortex may also facilitate neural rehabilitation by reducing astroglia scar formation.

BDNF is the most abundant neurotrophic factor in the brain. It is enriched primarily in the ipsilateral cortex and hippocampus of TBI. BDNF plays crucial roles in neurons’ survival, differentiation and axon regeneration throughout adulthood (Leibrock et al. 1989; Gustafsson et al. 2021). We reported that HSYA significantly upregulated BDNF expression in the cortex and hippocampus after TBI. The results agree with the enhanced neurogenesis and axon regeneration in these regions and coincide with former studies in vascular dementia (Xing et al. 2016).

STAT3 activation is essential for CNS axon regeneration (Leibinger et al. 2013; Mehta et al. 2016). Mechanically, the activated STAT3 binds to the promoter of GAP43 and facilitates its expression (Hung et al. 2016). The latter can promote axon outgrowth by directly binding to F-actin and modulating F-actin dynamics (Nguyen et al. 2009). A previous study indicates that HSYA could activate STAT3 to suppress Aβ-induced neuroinflammation (Zhang, Bogdanova, et al. 2014; Zhang, Yu, et al. 2014). We demonstrated that STAT3 was located in the centre of the neurogenesis and axon regeneration-specialized HSYA targets network of TBI. Moreover, HSYA significantly augmented GAP43 expression in the hippocampus and cortex following TBI. The results suggest that the activated STAT3/GAP43 axis probably medicates HSYA-induced post-TBI axon regeneration.

Taken together, this work indicated that HSYA promoted neurogenesis, facilitated axon regeneration and improved neurological deficits following TBI at the subacute stage. HSYA regulated ‘arginine biosynthesis,’ ‘arginine and proline metabolism’ pathways in the cortex and ‘phenylalanine, tyrosine and tryptophan biosynthesis’ pathways in the cortex and hippocampus. Also, HSYA enhanced BDNF expression and activated STAT3/GAP43 axis for neural restoration. The study uncovered the effects of HSYA on ‘blood stagnation syndrome’ at the subacute stage of TBI, revealed the mechanisms of HSYA on TBI recovery, and extended the potential effective windows of HSYA on TBI. However, These results were preliminary. More biological replicates, TBI models, animal species and clinical trials are needed to prove the efficacy and mechanisms of HSYA on post-TBI restoration. Moreover, the direct targets of HSYA merit further investigation.

## Supplementary Material

Supplemental Material

## Data Availability

The datasets in this study are available from the corresponding author upon reasonable request.
