# Supplementary material for: Metabolomics reveals the effects of hydroxysafflor yellow A on neurogenesis and axon regeneration after experimental traumatic brain injury
Source: Pharm Biol. 2023 Jul 7;61(1):1054–64. doi: 10.1080/13880209.2023.2229379 (PMC10332220; doi:10.1080/13880209.2023.2229379)
Supplement: Supplemental Material [file IPHB_A_2229379_SM3118.docx]

**Supplementary Table1**. Potential effectors of HSYA on neurogenesis and axon regeneration and the associated metabolites.

| Gene*^a^* | Score^b^ | Related metabolites^c^ |
| --- | --- | --- |
| *Bdnf* | 3.62 | L-(-)- Methionine, valine |
| *Stat3* | 3.18 | Argininosuccinic acid, L-(-)-Methionine, phloroglucinol |
| *Il1b* | 2.63 | L-(+)-Citrulline |
| *Th* | 2.44 | Ascorbic acid, L-Phenylalanine |
| *Slc6a3* | 2.43 | ecgonine, valine |
| *Il6* | 1.82 | trans-3-Indoleacrylic acid, Ascorbic acid, L-(+)-Citrulline, ornithine, L-(-)-Methionine, Pantothenic acid, L-Phenylalanine, phloroglucinol, N3, N4-Dimethyl-L-arginine, thiamine, valine |
| *Vegfa* | 1.66 | Ascorbic acid, L-(+)-Citrulline, D-(+)-Proline, L-(-)-Methionine, Pantothenic acid, phloroglucinol, N3, N4-Dimethyl-L-arginine |
| *Tp53* | 1.54 | 2'-Deoxyinosine, Ascorbic acid, L-(+)-Citrulline, Argininosuccinic acid, L-(-)-Methionine, Pantothenic acid, L-Phenylalanine, phloroglucinol, proline, thiamine, valine |
| *Nos2* | 1.52 | Ascorbic acid, L-(+)-Citrulline, Argininosuccinic acid, ornithine, N3, N4-Dimethyl-L-arginine, Pantothenic acid, phloroglucinol |
| *Slc6a4* | 1.5 | DL-Tryptophan, valine |
| *Ptgs2* | 1.15 | DL-Dipalmitoylphosphatidylcholine, Ascorbic acid, L-(+)-Citrulline, phloroglucinol, N3, N4-Dimethyl-L-arginine, thiamine |
| *Pparg* | 1.03 | Ascorbic acid |
| *Pik3ca* | 0.98 | Argininosuccinic acid, L-(-)-Methionine, phloroglucinol |
| *Ins2* | 0.95 | trans-3-Indoleacrylic acid, Ascorbic acid, L-(+)-Citrulline, D-(+)-Proline, Argininosuccinic acid, L-Norleucine, ornithine, L-(-)-Methionine, Pantothenic acid, L-Phenylalanine, proline, N3, N4-Dimethyl-L-arginine, thiamine, valine |
| *Mtor* | 0.87 | L-(+)-Citrulline, Argininosuccinic acid, L-Norleucine, ornithine, L-(-)-Methionine, L-Phenylalanine, valine |
| *Kdr* | 0.84 | L-(-)-Methionine |
| *Nfkb1* | 0.66 | Ascorbic acid |
| *Casp3* | 0.65 | Ascorbic acid, DL-Tryptophan, L-(-)-Methionine, phloroglucinol, thiamine |
| *Insr* | 0.59 | D-(+)-Proline, L-Phenylalanine, valine |
| *Ak2* | 0.55 | pipecolic acid |
| *Anxa5* | 0.55 | Ascorbic acid, Argininosuccinic acid, phloroglucinol |
| *Car2* | 0.55 | ornithine, phloroglucinol |
| *Cat* | 0.55 | Ascorbic acid, L-(+)-Citrulline, DL-Tryptophan, D-(+)-Proline, Argininosuccinic acid, ornithine, pipecolic acid, L-(-)-Methionine, Pantothenic acid, L-Phenylalanine, phloroglucinol, proline, thiamine, valine |
| *Fh* | 0.55 | Argininosuccinic acid |
| *Mapk8* | 0.55 | Ascorbic acid, phloroglucinol |
| *Nos3* | 0.55 | Argininosuccinic acid, Ascorbic acid, L-(+)-Citrulline, ornithine, N3, N4-Dimethyl-L-arginine |
| *Tf* | 0.55 | Ascorbic acid, ecgonine, ornithine, L-(-)-Methionine, Pantothenic acid, thiamine |
| *Hif1a* | 0.46 | Ascorbic acid, Argininosuccinic acid, proline |
| *Mmp9* | 0.46 | D-(+)-Proline, N3, N4-Dimethyl-L-arginine |
| *Bace1* | 0.45 | phloroglucinol |
| *Cck* | 0.38 | 2'-Deoxyinosine, D-(+)-Proline, L-Norleucine, L-(-)-Methionine, L-Phenylalanine |
| *Cdh1* | 0.36 | L-(-)-Methionine |
| *Ang* | 0.32 | DL-Dipalmitoylphosphatidylcholine, D-(+)-Proline, L-(-)-Methionine, phloroglucinol, N3, N4-Dimethyl-L-arginine |
| *Cckbr* | 0.31 | 2'-Deoxyinosine, L-Norleucine |
| *Il4* | 0.22 | trans-3-Indoleacrylic acid, L-(+)-Citrulline, ornithine, pipecolic acid, N3, N4-Dimethyl-L-arginine |
| *Mapk14* | 0.22 | Ascorbic acid, phloroglucinol, proline |
| *Tlr4* | 0.22 | phloroglucinol |
| *Bcl2* | 0.19 | Ascorbic acid, L-(-)-Methionine, phloroglucinol |
| *Il10* | 0.19 | trans-3-Indoleacrylic acid, Ascorbic acid, L-(+)-Citrulline, N3, N4-Dimethyl-L-arginine |
| *Hmox1* | 0.14 | Ascorbic acid, thiamine |
| *Plat* | 0.14 | DL-Dipalmitoylphosphatidylcholine, L-Norleucine |
| *Casp9* | 0.1 | phloroglucinol |
| *Jak2* | 0.1 | L-Phenylalanine |

^a^: The shared genes in the differential metabolite-related genes, the neurogenesis and axon regeneration-related genes, and HSYA related genes. ^b^: The relevance score of the gene to neurogenesis and axon regeneration. ^c^: The metabolites related to the genes.
